# Optically detected nuclear magnetic resonance of coherent spins in a molecular complex

**DOI:** 10.1038/s41563-026-02539-0

**Published:** 2026-03-19

**Authors:** Evgenij Vasilenko, Vishnu Unni Chorakkunnath, Jeremias Resch, Nicholas Jobbitt, Diana Serrano, Philippe Goldner, Senthil Kumar Kuppusamy, Mario Ruben, David Hunger

**Affiliations:** 1 Institute for Quantum Materials and Technologies (IQMT), Karlsruhe Institute of Technology, Karlsruhe, Germany; 2 Physics Institute (PHI), Karlsruhe Institute of Technology, Karlsruhe, Germany; 3 Chimie ParisTech, PSL University, CNRS, Institut de Recherche de Chimie Paris, Paris, France; 4 Institute of Nanotechnology (INT), Karlsruhe Institute of Technology, Karlsruhe, Germany; 5 Centre Européen de Sciences Quantiques (CESQ), Institut de Science et d’Ingénierie Supramoléculaires (ISIS), Strasbourg, France

**Keywords:** Quantum information, Optical materials and structures

## Abstract

Nuclear magnetic resonance is a powerful tool for applications ranging from chemical analysis to quantum information processing. Achieving optical initialization and detection of molecular nuclear spins promises new opportunities—including improved nuclear magnetic resonance signals at low magnetic field, sensitivity down to the single-molecule level and full access to atomically precise molecular architectures for quantum technologies. Here we report the optical read-out of coherently controlled nuclear spins in a europium-based molecular crystal. By harnessing ultranarrow optical transitions, we achieve the optical initialization and detection of nuclear spin states. Through radio-frequency driving, we address two nuclear quadrupole resonances, characterized by narrow inhomogeneous linewidths and a distinct correlation with the optical transition frequency. We implement Rabi oscillations, spin echo and dynamical decoupling techniques, achieving nuclear spin quantum coherence with a lifetime of up to 2 ms. These results highlight the capabilities of optically detected nuclear magnetic resonance and underscore the promise of molecular nuclear spins for quantum information processing.

## Abstract (web summary)

Optical initialization and read-out of nuclear spins with a spin coherence time of 2 ms in a Eu^3+^ molecular crystal is demonstrated with radio-frequency control, providing coherent nuclear spin manipulation for quantum information technologies.

## Main

Nuclear magnetic resonance (NMR) is a well-established and highly developed field that plays a vital role across a wide range of applications—from pharmaceutical quality control to materials research. Due to their weak interaction with the environment, nuclear spins are a valuable resource for quantum technology^1,2^ that may allow for dense qubit registers operable at a comparably high temperature. Optical addressing of nuclear spins serves as an important tool to leverage them as qubits for quantum memories^3–5^ and quantum processors^6,7^. Nuclear spin addressing is usually achieved only indirectly^8^ through optical transitions linked to electron spins. Such addressing is successfully used in colour centres in diamond^6,7,9,10^, silicon carbide^11,12^ and semiconductor quantum dots^13,14^. Optically addressable molecular spin qubits^15,16^ offer a novel platform in this context, where optical and spin properties can be tailored, photonic integration is facilitated^17^ and supramolecular assemblies may enable atomically precise qubit registers^18^. Early pioneering work has introduced optically detected magnetic resonance in molecular spins down to the single-molecule level^19,20^, and recent results based on transition metal complexes^21,22^, organic radicals^23,24^ and lanthanide complexes^25–27^ have spurred new interest.

Indirect optical addressing of nuclear spins relies on magnetic coupling between the electron and nuclear spins. This coupling is typically weak and, thus, constrains the bandwidth for spin initialization, manipulation and read-out. Furthermore, the presence of electron spin typically introduces noise and limits the coherence of coupled nuclear spins^28^. The strong magnetic moment of electron spins also limits their useful density to avoid undesired couplings^29^.

Trivalent non-Kramers rare-earth ions such as Eu^3+^ and Pr^3+^ represent a notable exception by offering directly addressable nuclear spins. They possess no net electronic spin and offer narrow optical transitions that enable direct resolution, initialization, manipulation and read-out of nuclear spin states^30^. For example, in europium-doped yttrium orthosilicate (Eu^3+^:Y_2_SiO_5_) crystals, optically detected nuclear spin quantum coherence with a lifetime exceeding 10 h has been demonstrated^31^, and the quantum storage of photons in nuclear spin states for up to 1 h has been achieved^4^. Recently, it has been shown that europium-based molecular complexes show outstanding optical coherence properties and long nuclear spin lifetimes, which allow for direct optical nuclear spin access and optical spin initialization^25,26^. However, combining optical addressing and coherent nuclear spin control has remained elusive for molecular complexes so far.

In this work, we demonstrate the optical initialization and read-out of coherently controlled nuclear spins in a europium-based molecular complex, marking an important advancement towards establishing this system as a viable platform for quantum technologies.

**Fig. 1:**
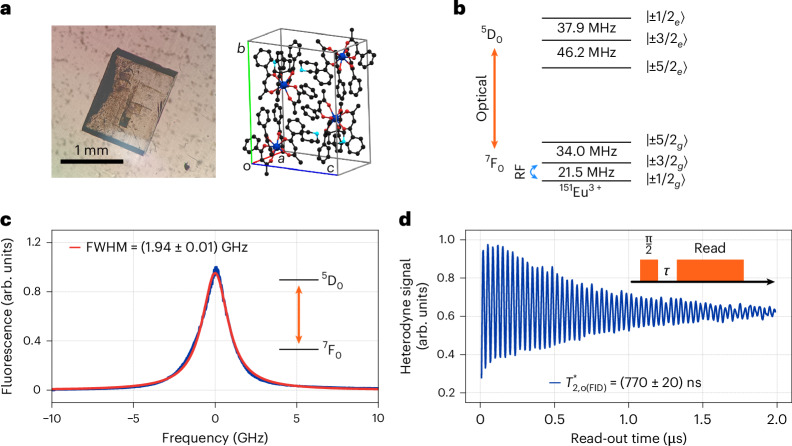
Molecular crystal and optical properties. **a**, Molecular structure of the complex and crystal unit cell obtained from single-crystal X-ray diffraction. Blue, europium; red, oxygen; black, carbon; cyan, nitrogen; hydrogen is omitted for clarity. Single crystals with ~1-mm size and clear facets were grown. **b**, Ground- and excited-state hyperfine levels of the ^7^F_0_ → ^5^D_0_ transition of ^151^Eu^3+^. **c**, Photoluminescence excitation measurement of the ^7^F_0_ → ^5^D_0_ transition showing a narrow inhomogeneous linewidth of 1.94(1) GHz. FWHM, full-width at half-maximum. **d**, Optical FID of the optical transition dipoles of ions. Coherent oscillations can be observed as a beating signal via heterodyne detection with a frequency-detuned optical read-out pulse. A fit to the signal yields a pure dephasing time of ${T}_{2,{\rm{o}}({\rm{FID}})}^{* }=$ 770(20) ns.

We study stoichiometric molecular crystals composed of a mononuclear Eu^3+^ complex [Eu(BA)_4_(pip)], where BA and pip refer to benzoylacetonate and piperidinium, respectively. The complex comprises an eight-coordinated anionic fragment ${[{{\rm{Eu(BA)}}}_{{\rm{4}}}]}^{-}$, and the charge-balancing piperidinium cation. The complex crystallizes in a monoclinic lattice with four molecules per cell (Fig. 1a). A detailed optical characterization of the europium complex in a microcrystalline powder^25^ has evidenced narrow optical homogeneous linewidths connected to the ^7^F_0_ → ^5^D_0_ transition, as well as optical spin polarization and long-lived nuclear spin states, which have made it possible to infer the energy-level structure (Fig. 1b). To optimize the homogeneity of the material, we have grown millimetre-sized molecular crystals via slow solvent evaporation (Fig. 1a; also see the ‘Growth of molecular crystals’ section in the Supplementary Information). For optical read-out, we incorporate a crystal into a fibre-based ferrule setup (see the ‘Optical setup’ section in the Supplementary Information). The setup is directly immersed in liquid helium, providing effective thermalization and a stable temperature of 4.2 K. In addition, a superconducting coil is installed to address the hyperfine transitions by applying radio-frequency (RF) fields.

As the first step, we characterize the optical properties to assess the impact of crystal quality. We scan a tunable dye laser across the ^7^F_0_ → ^5^D_0_ transition and observe an inhomogeneous linewidth of *Γ*_inh_ = 1.94(1) GHz (Fig. 1c). This is more than factor-of-three narrower compared with the value (6.6 GHz) reported for a microcrystalline powder in a previous study^25^, evidencing a reduced amount of structural defects in the crystal. To access the homogeneous linewidth *Γ*_h_, we performed spectral hole burning (SHB) measurements at low laser powers of 20 μW to avoid power broadening. The narrowest observed hole width is 620(60) kHz (Supplementary Fig. 1), corresponding to a homogeneous linewidth of *Γ*_h_ = 310 kHz and a coherence time of ${T}_{2,{\rm{o}}}^{* }=1/({{\pi }}{\varGamma }_{{\rm{h}}})=1.03(10)\,\mu s$.

Although SHB probes the long-term homogeneous linewidth, a measurement of the optical free-induction decay (FID) gives access to the instantaneous pure dephasing time ${T}_{2,{\rm{o}}}^{* }$ (ref. ^32^). The pulse sequence consists of an optical π/2 pulse that creates optical coherence and a frequency-shifted read-out pulse that interferes with the radiated field for heterodyne detection. The decaying beating signal yields a lower limit for the dephasing time of ${T}_{2,{\rm{o}}({\mathrm{FID}})}^{* }=0.770(20)\,{{\upmu} s}$ (Fig. 1d). This is slightly shorter than the SHB measurement, indicating that the elevated power required for the π/2 pulse in the FID measurement leads to power broadening that dominates over spectral diffusion^32^. To probe the optical coherence time *T*_2,o_, two-pulse photon echo experiments with heterodyne detection were performed, which yield *T*_2,o_ = 2.13(3) μs. This value is also improved compared with the reported coherence time (1.49 μs) at this temperature^25^, which is mainly limited by phonon-induced dephasing (see the ‘Optical dephasing’ section in the Supplementary Information). Overall, this demonstrates that high-quality single crystals lead to improved inhomogeneous and homogeneous optical linewidths compared with microcrystalline samples.

**Fig. 2:**
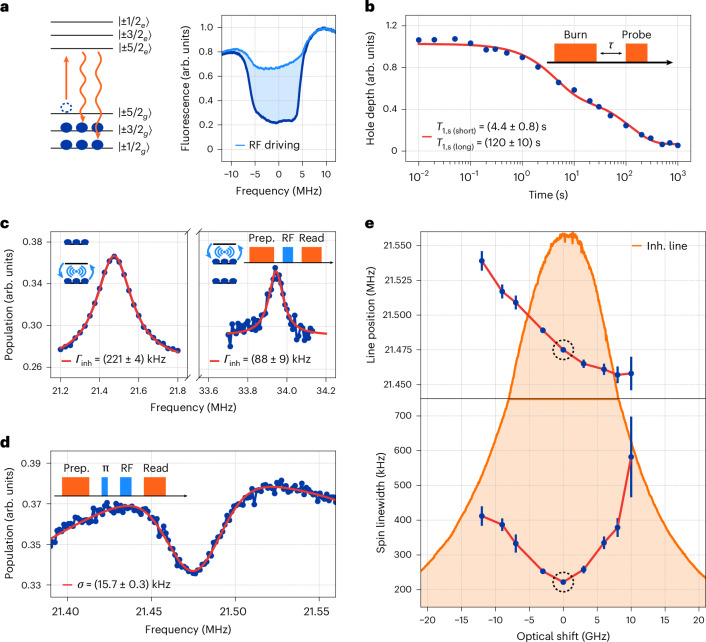
Optically detected NMR and spin state lifetime. **a**, Schematic of the depopulation of a hyperfine level by optical pumping for spin state preparation (left). A spectral pit of 10-MHz width is prepared (right; dark blue), and resonant RF driving repopulates the level and fills the pit (light blue). **b**, Time evolution of the pit depth is fitted with a double-exponential decay, resulting in two spin relaxation times: *T*_1,s(short)_ = 4.4 s and *T*_1,s(long)_ = 122 s. **c**, Pointwise measurement of an ODNMR spectrum for the two ground-state transitions at 21.475 MHz (left) and 33.944 MHz (right). The spin transition has an inhomogeneous linewidth of 221(4) kHz and 88(9) kHz, respectively. **d**, Spin hole burning spectrum to probe a homogeneous class of spin transition. An RF π pulse is applied to burn a spin hole before the ODNMR measurement is performed. The observed hole exhibits a linewidth of 15.70(30) kHz. **e**, Measurement of the centre frequency (top) and the spin inhomogeneous (Inh.) linewidth (bottom) of the 21.5-MHz transition as a function of optical frequency. The dashed circles mark the spectral position of all other measurements. Each data point corresponds to a single independent measurement of the spin inhomogeneous line (*n* = 1 per data point), with central values and uncertainties derived from the fitting procedure. The optical inhomogeneous line is shown in orange for comparison.

We now turn to optical spin initialization and the detection of spin resonances. A spectral pit is created by optically pumping ions out of a selected hyperfine ground state, thereby depleting its population and producing a transparency window in the absorption profile. Although the preparation of a sub-ensemble within a single hyperfine state can be achieved with a series of optical pulses^33^, we find that sufficient signal contrast is achieved by burning a single spectral pit of 10-MHz width with a laser chirp, which depopulates one hyperfine level for a certain ion class (Fig. 2a). This is achieved faster than full class preparation, and is used in all subsequent experiments for initial spin state preparation. We measure the spin lifetime *T*_1,s_ by probing the depth of the pit as a function of the waiting time. Figure 2b shows the decay of the pit depth over time, yielding two characteristic time constants: *T*_1,s(short)_ = 4.4(8) s and *T*_1,s(long)_ = 120(10) s (see the ‘Nuclear spin lifetime’ section in the Supplementary Information). In particular, *T*_1,s(short)_ is one order of magnitude longer than reported previously^25^, despite a higher temperature (4.2 versus 1.5 K).

We focus on the ground-state nuclear quadrupole transitions of the isotope ^151^Eu^3+^ within a natural abundance sample, where the transition frequencies were previously estimated from SHB^25^ (Fig. 1b). We perform optically detected NMR (ODNMR) measurements for the precise determination of transition frequencies and to probe the spin inhomogeneity. Therefore, after spin polarization, a weak optical probe pulse is applied, where we sweep the laser frequency by 15 MHz over the spectral pit to read out the resulting fluorescence signal as a reference. Afterwards, a 1-ms RF pulse at a constant frequency with a power of ~92 W is applied with the coil. The resulting change in spin population is read out with a final optical pulse to measure the fluorescence in the middle of the pit. Since the RF pulse addresses only a subset of the depleted hyperfine levels within the optically resonant ion classes, it repopulates only a fraction of the spectral pit (Fig. 2a). We average the signal over five repetitions and, subsequently, change the RF frequency before repeating the sequence to probe the spin transition point by point. Figure 2c shows both ground-state resonances with centre frequencies of 33.944(2) MHz ($\left|3/2\right\rangle \leftrightarrow \left|5/2\right\rangle$) and 21.475(1) MHz ($\left|1/2\right\rangle \leftrightarrow \left|3/2\right\rangle$).

The spin inhomogeneous line at 34 MHz has a Lorentzian shape with a full-width at half-maximum of 88 kHz, similar to high-quality europium-doped solid-state crystals^30,34^, and almost factor-of-three narrower than the 21.5-MHz transition. The latter transition shows a much larger signal contrast for the same pulse parameters, indicating a larger transition strength. We observe a power-dependent broadening of the 21.5-MHz transition and find a linewidth of 154 kHz at the lowest power of 0.5 W. We can estimate the homogeneous spin linewidth by performing spin hole burning. Therefore, we use the sequence for ODNMR and apply an additional RF π pulse to remove a resonant class of spins from the probed ensemble. This produces a narrow hole in the spin inhomogeneous line (Fig. 2d), and a Gaussian fit yields a hole width of 15.70(32) kHz, which reduces to 11,64(60) kHz when minimizing the RF burning power, corresponding to a lower bound of the spin dephasing time ${T}_{2,{\rm{s}}}^{* }=19.3\,\upmu \rm{s}$.

The crystal used for spin characterization has an optical inhomogeneous linewidth of 23 GHz, indicating larger strain in the crystal, possibly due to mechanical forces during insertion into the ferrule setup. It is, thus, interesting to investigate the dependence of the spin transition properties across the optical inhomogeneous line. We, therefore, measure the line position and the inhomogeneous linewidth of the 21.5-MHz transition (Fig. 2e). We observe a nonlinear dependence of the spin transition frequency with the optical probing frequency, which can be approximated with a linear gradient of −4 kHz GHz^−1^. This shows an opposing sign and a smaller magnitude compared with the value of 10 kHz GHz^−1^ reported for solid-state crystals^35^. We ascribe these differences to the presence of a crystal field asymmetry parameter of *η* = 0.47, and to the *C*_2*v*_ point group symmetry of Eu^3+^ in our complex (see the ‘Correlation between optical and spin inhomogeneous lines’ section in the Supplementary Information), which both differ compared with ref. ^35^. This indicates differences in the contribution of symmetry-dependent crystal field parameters, which can change both magnitude and sign of the correlation gradient between optical and RF transitions. In addition, the comparably large optical inhomogeneous linewidth of the crystal studied for these measurements suggests a broader variation range of the quadrupolar parameters, making it plausible that a linear approximation is insufficient. Furthermore, we observe a notable increase in spin inhomogeneous broadening towards the wings of the optical line. This indicates that high crystalline quality also reflects in narrow-spin inhomogeneous lines, and that strain affects optical and spin transitions in a correlated manner specific for the respective ligand field. These measurements exemplify the potential of ODNMR for studying materials properties and for applications such as strain or pressure sensing^36^.

**Fig. 3:**
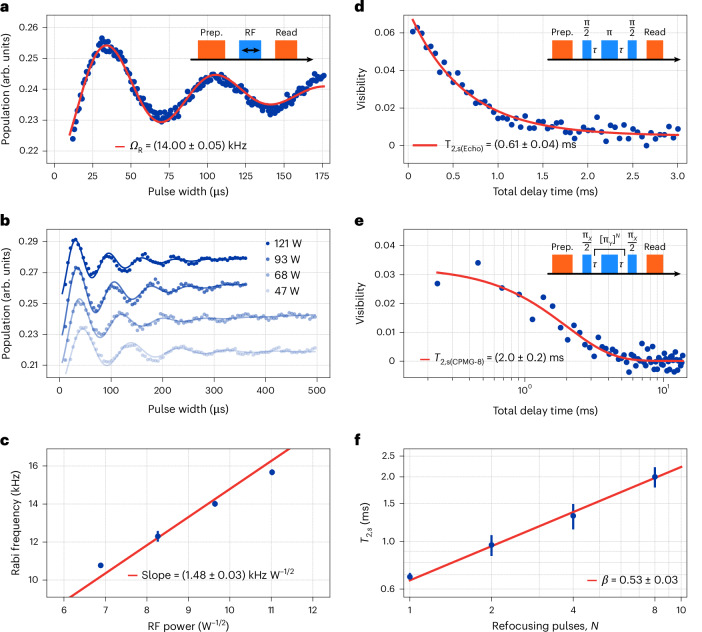
Coherent nuclear spin manipulation and spin coherence time. **a**, Rabi oscillations observed by varying the RF pulse duration, revealing a Rabi frequency of 14 kHz. The oscillations involve a damping component resulting from inhomogeneous broadening. **b**, The Rabi frequency increases with the RF power. For improved visualization, the individual curves are vertically offset by constant values. **c**, Power dependence of the Rabi frequency shows the expected scaling with a proportionality factor of 1.48(3) kHz W^−1/2^. **d**, Hahn-echo sequence is used to probe the spin coherence *T*_2,s_. The decay of the echo signal yields *T*_2,s_ = 613 μs. **e**, Spin coherence time is extended to 2 ms by CPMG dynamical decoupling using eight decoupling pulses. **f**, CPMG measurement was conducted for *N* = 1, 2, 4 and 8 refocusing pulses, showing an increase in coherence time by a factor of *N*^*β*^, where *β* = 0.53(3). In **c** and **f**, each data point represents a single Rabi and CPMG measurement, respectively (*n* = 1 per data point). The displayed central values and uncertainties are obtained from fits to the corresponding datasets.

Finally, we harness the optical spin initialization and read-out to investigate coherent nuclear spin control. As a demonstration, we perform nuclear Rabi oscillations, and adapt the pulse sequence for ODNMR by choosing a resonant RF frequency to match the 21.5-MHz transition and vary the pulse length in steps of 1 μs. Figure 3a shows the resulting Rabi oscillations, which reveal a Rabi frequency *Ω*_R_ = 14 kHz at an RF power of 92 W. The damping of the oscillation originates from the inhomogeneity of the transition. We repeat this measurement for different RF powers and observe oscillations with increasing Rabi frequency (Fig. 3b). We find a power dependence that follows the expected square root law ${{\Omega }}_{{\rm{R}}}\propto \sqrt{P}$ with a proportionality factor of 1.48 kHz W^−1/2^ (Fig. 3c).

The inhomogeneous broadening of the spin transition as well as slow fluctuations of the local magnetic field lead to dephasing that can be compensated by pulsed NMR sequences. We implement a Hahn-echo sequence, using a π-pulse duration of 36 μs for *Ω*_R_ = 14 kHz as obtained from Rabi oscillations. To obtain a quantitative contrast, the sequence is performed twice, once with and once without a phase shift of 180° of the final π/2 pulse. The difference between the two measurements is normalized to their sum and referred to as the visibility. Figure 3d shows an example dataset of the visibility as a function of the delay time *τ*, yielding an exponential decay with a time constant corresponding to the coherence time *T*_2,s_ = 0.61(4) ms. This value is comparable with the spin coherence observed in high-quality europium-doped solid-state crystals^30^, and underlines the promising properties of this molecular material.

To further protect the nuclear spins from decoherence, dynamical decoupling control using Carr–Purcell–Meiboom–Gill (CPMG) sequences was applied. Such protection can, under certain circumstances, strongly increase the spin coherence time, as this sequence also compensates for pulse imperfections. This is achieved by applying the refocusing π pulses along a 90°-rotated axis compared with the π/2 pulses. CPMG measurements were performed for *N* = 1, 2, 4 and 8 refocusing pulses. Figure 3e depicts a representative measurement for *N* = 8 pulses, for which we could observe a spin coherence time of up to 2.0(2) ms. The coherence time follows a scaling law described by

1 $${T}_{2,{\rm{s}}(\mathrm{CPMG})}={T}_{2,{\rm{s}}(\mathrm{Echo})}\times {N}^{\beta },$$

where *β* denotes the scaling factor and *T*_2,s(Echo)_ denotes the coherence time obtained from the Hahn-echo measurements. All visibility decays were fitted with stretched exponential functions (stretching factors of 1.43, 1.18, 1.32 and 1.31 for one, two, four and eight π pulses, respectively). The results (Fig. 3f) demonstrate that dynamical decoupling considerably improves the spin coherence time, with *β* = 0.53 ± 0.03, which is slightly off the 2/3 scaling expected for a correlated noise bath of a single spin species^37,38^. The scaling shows no saturation, such that increasing the number of refocusing pulses could extend the coherence time further. The observed stretching factors agree with the value of 1.5 obtained for spin ensembles^39–41^. Using this ensemble-averaged model together with the measured bath coupling strength of *b* ≈ 12 kHz obtained from fitting the most-narrow spin hole observed with a Gaussian function yields an estimate of the bath correlation time *τ*_B_ ≈ 5.5 ms. The observed CPMG scaling exponent *β* indicates that the nuclear spins interact with a heterogeneous environment rather than a single Lorentzian noise bath. This non-trivial environment probably arises from a combination of nearby proton spins; randomly distributed ^13^C spins; residual paramagnetic impurities originating from europium salt precursor (purity level, 99.99%), such as Gd^3+^, Nd^3+^ and Dy^3+^; and quasi-localized low-frequency vibrational modes (see the ‘Nuclear spin dephasing sources’ section in the Supplementary Information).

In summary, our results have shown direct access to coherently controlled nuclear spins in a molecular material. Due to the weak magnetic moment of the europium nuclear spin, a long spin coherence could be observed, and even longer coherence is expected by applying more decoupling pulses and operating at millikelvin temperatures in a magnetic field to polarize paramagnetic impurities and freeze low-frequency vibrational modes. Also, purification and chemical engineering of the complex (for example, by deuteration) is expected to improve nuclear spin coherence. Furthermore, optical addressing of nuclear spins may open a new avenue into NMR-based materials characterization. Super-hyperfine coupling to neighbouring spins may offer unique signatures that could allow for structure analysis^42,43^, enable the polarization of ligand-centred nuclear spins and possibly their full quantum control. Furthermore, the coherent optical transitions are a powerful tool to achieve direct optical spin manipulation^34,44^. This holds great promise for realizing fast spin qubit control including single- and two-qubit gates^45^. When studied at the single-molecule level (for example, by integration into nanophotonic cavities^17,46,47^), optically addressable nuclear spins in molecules offer a promising route to realize atomically precise multiqubit quantum registers for scalable and optically connectable quantum processing nodes^47,48^.

### Reporting summary

Further information on research design is available in the Nature Portfolio Reporting Summary linked to this article.

## Online content

Any methods, additional references, Nature Portfolio reporting summaries, source data, extended data, supplementary information, acknowledgements, peer review information; details of author contributions and competing interests; and statements of data and code availability are available at 10.1038/s41563-026-02539-0.

## Supplementary information

Supplementary Information Supplementary Figs. 1–4, Table 1, materials and methods, and theoretical considerations.

Reporting Summary

## Data Availability

All data generated and analysed during this study are available via Code Ocean at 10.24433/CO.4566380.v1.

## Code availability

The custom code used for data analysis is available via Code Ocean at 10.24433/CO.4566380.v1.
