# Supplementary material for: Optically detected nuclear magnetic resonance of coherent spins in a molecular complex
Source: Nat Mater. 2026 Mar 19;25(7):1154–9. doi: 10.1038/s41563-026-02539-0 (PMC13323051; doi:10.1038/s41563-026-02539-0)
Supplement: Supplementary file 1 — Supplementary Figs. 1–4, Table 1, materials and methods, and theoretical considerations. [file 41563_2026_2539_MOESM1_ESM.pdf]

nature materials

Supplementary information

https://doi.org/10.1038/s41563-026-02539-0

Optically detected nuclear magnetic resonance of coherent spins in a molecular complex

In the format provided by the authors and unedited

Contents

1 Materials and Methods **S2**
1.1 Growth of molecular crystals S2
1.2 Optical setup S2
1.3 RF setup S3
1.4 Spectral pit preparation S4
1.5 Data normalization and fitting procedures S4

2 Theoretical considerations **S6**
2.1 Addressable ions in a spin measurement S6
2.2 Optical dephasing S6
2.3 Nuclear spin lifetime S7
2.4 Nuclear spin dephasing sources S7
2.5 Correlation between optical and spin inhomogeneous lines S9

3 Figures **S11**

4 Tables **S13**

References **S14**

1 Materials and Methods

1.1 Growth of molecular crystals

The all-oxygen coordination environment of the Eu^{3+} optical center is described as biaugmented trigonal prism and exhibits a C_{2v} point group symmetry. We grow macroscopic molecular crystals by using the method of slow solvent evaporation in order to produce millimeter-sized large molecular crystals. In this method, the crystalline powder sample is dissolved in a small amount (100 mg in 6 mL) of ethanol to reach a near-saturated solution. The solvent slowly evaporates over time, while macroscopic crystals start to form and slowly grow. Depending on the solvent evaporation rate, the quality of the crystal varies drastically. A rough control of the evaporation rate is achieved by perforating the covering foil of the beaker with the solution. We present results on two different macroscopic crystals. The first crystal was grown with a solvent evaporation time close to three weeks. This crystal has lateral dimensions of about 1 mm × 1 mm and a thickness of approximately 200 µm. The crystal was integrated into a dilution refrigerator. In order to provide efficient thermalization, the crystal was glued onto a copper sheet and positioned on a copper mount for free space excitation with a confocal microscope. Only the inhomogeneous linewidth was measured for this crystal under these conditions. The second crystal was recrystallized within a time period of four days with dimensions of 1.5 mm × 1.5 mm and a thickness of about 500 µm. This crystal was integrated into a fiber-based ferrule setup, and directly immersed in liquid helium, providing effective thermalization and a stable temperature of 4.2 K. All other measurements shown were preformed with the second crystal under these conditions.

A superconducting coil, which is described in detail in Subsection **RF setup**, was installed around the ferrule setup and combined with an LC-circuit to enable the addressing of both hyperfine transitions of the molecular crystals (see Fig. 1.3a). Both crystals were grown from dissolved microcrystalline powder with natural abundance of the Europium isotopes with 100 % Eu^{3+} ion doping concentration. All measurements were performed on the $^{151}Eu^{3+}$ isotope, since the reported hyperfine splittings shown in Fig. 1b are smaller compared to the other isotope [1], and are therefore accessible with the frequency range provided by our setup.

1.2 Optical setup

For both cryostats used, the same optical setup could be employed. The molecular samples were resonantly excited by a Sirah Matisse 2DX dye laser, which exhibits a FWHM linewidth of $<$ 50 kHz and can be mode-hop-free tuned over (∼75 GHz). The wavelength for all experiments was adjusted to approximately 580.377 nm to match the coherent $^7F_0 \rightarrow {}^5D_0$ transition. The main laser beam is divided into two parts: one beam is used as a reference path, as the laser output is monitored via an optical spectrum analyzer (OSA, Bristol Instruments 771 Series) to verify the laser frequency and single-mode operation, while the other beam is guided through an acousto-optic modulator (AOM, Gooch&Housego 3200-121) in the double-pass configuration enabling the shaping and control of optical pulses in amplitude and frequency. The AOM is

controlled by an arbitrary waveform generator (AWG, Quantum Machnies OPX+). After the AOM, the light is fiber-coupled and can be directed either to the dilution refrigerator or a home-built dipstick cryostat which incorporates the fiber-based ferrule setup. The current ferrule setup consists of two opposing small cylindrical ferrules with a diameter of 2.5 mm, one made out of ceramic and the other out of Teflon. The ceramic ferrule has a bore size of 300 µm which is employed to integrate the excitation multimode fiber with a core diameter of 200 µm. Since no ceramic ferrules with bore sizes of 1.5 mm are commercially available, we machined a Teflon ferrule to incorporate a multimode fiber with a core diameter of 1000 µm. This large core fiber was pulled back by about 0.7 mm, introducing enough sample space for large-scaled crystalline samples. Both ferrules were put in a commercial ceramic mating sleeve connector and glued to provide more stability of the setup. In the dilution refrigerator, the macroscopic crystal was placed onto a copper mount and excited in a free-space geometry. The signal transmitted through the crystal was collected through a window of the cryostat. In both cases, the resulting signal was detected by a Thorlabs avalanche photodetector (APD, APD130A2/M). To enable selective detection of optical signals from the sample, additional filters can be employed. A longpass filter (BLP01-594R-25) is used to block the excitation light and observe fluorescence, while a bandpass filter (FB580-10) with a spectral range of ±10 nm is used for detecting absorption around the resonantly excited $^7F_0 \rightarrow {}^5D_0$ transition.

1.3 RF setup

The RF signal is produced by the same AWG used for the creation of the optical pulses, allowing precise control and synchronization between applied optical and RF pulses. The RF signal is guided through an 10 dB attenuator to prevent damage from potential reflections, and then amplified using a high-power RF amplifier (Bonn Elektronik, BSA 1025-150) capable of delivering up to 150 W of output power. The amplified signal is coupled into the cylindrical RF coil via a capacitor-based 50 Ω matching circuit, which allows tuning of the resonance frequency, bandwidth, and depth of the resonance through adjustable parallel and series capacitors (a few tens of pF). Before a measurement, a vector network analyzer (VNA, E5071B ENA) is connected to this LC-circuit in order to monitor the resonance profile and to adjust the resonance frequency to the specific hyperfine transition. On the output side of the RF coil, a high-power 30 dB attenuator is placed to reduce these high powers significantly, which is followed by additional attenuators and a 50 Ω termination connector to suppress reflections and ensure impedance matching.

For the measurements presented in this work, a home-built cylindrical coil was used. The coil features closely spaced turns without air gaps and is encapsulated in epoxy adhesive to ensure mechanical stability. The following paragraph summarizes its key characteristics in detail.

- **Wire material**: copper-stabilized superconducting wire from SUPERCON (type 54S43) and diameter of 0.43 mm (insulated)
- **Geometry:** see Fig. S2a and Table S1

- **Characterization of resonance:** see Fig. S2b, FWHM of 1.9 MHz and quality factor $Q{\sim}11$.

1.4 Spectral pit preparation

The spin characterization of the complex requires spin polarization of the $^{151}Eu^{3+}$ nuclei. A general spin initialization by preparing a spectral pit is carried out in this work. The lifetime of the spectral pit is limited by spin $T_{1,s}$, which is orders of magnitude longer than the spin characterization measurement sequence time. The laser frequency is swept by 10 MHz around the peak of the optical inhomogeneous lines within 300 ms. This is repeated 20 times to prepare a spectral pit with a contrast of about 60 %. An exemplary preparation sequence is shown in Fig. S3a. The prepared spectral pit is shown in Fig. S3b. This spectral pit contains nine classes of $^{151}Eu^{3+}$ ions out of which six contribute to the spin signal when driven resonantly. After the pulse sequences for spin driving, all of which are finished within 1 ms to 15 ms, a series of optical pulses with a frequency chirp over 100 MHz within 300 ms is applied 20 times to restore the spin states of $^{151}Eu^{3+}$ ions to their initial distribution.

1.5 Data normalization and fitting procedures

Fluorescence and transmission SHB signals:

Each SHB measurement consists of two measurements: one without a burning pulse to record the background, and one with the burning pulse for the SHB signal. The background trace contains a transient signal caused by the AOM. To correct for this, the SHB trace is divided by the background trace, which ideally results in a baseline at 1. The hole contrast in fluorescence or transmission should then be obtained relative to this baseline and should therefore be ≤ 1. In practice, slight mismatches between the background and SHB traces can occur and may shift the baseline above or below 1, typically due to laser instabilities such as frequency drifts or power fluctuations. Fig. S1a shows a spectral hole measurement with a slightly shifted baseline, nevertheless, the transmission contrast of the transmission signal is approximately 8 % and does not exceed 1.

Heterodyne signal:

The heterodyne signal is measured as the beating between the incoming signal field and a frequency-shifted local oscillator field, detected on a fast APD. The resulting oscillation appears on top of the heterodyne readout pulse, as visualized on an oscilloscope, and the measured beat amplitude is directly given by the APD voltage.

Spin population signal:

For the spin measurements, we introduced the quantity *population*, which is directly obtained from the fluorescence signal recorded with the APD. The probe pulse is integrated over its full temporal extent and normalized to the mean value of the central part of the final pit burn pulse, which serves as a background reference in all measurements. Because the fluorescence from the probed ion ensemble is very strong,

a single-photon counting module is not required, instead, the APD output voltage is used to represent the spin population signal.

Spin lifetime fitting and analysis:

In Fig. 3b, the two time constants are less visually pronounced than in Serrano et al. [1], but they are not an artefact of the fitting procedure. A mono-exponential fit does not adequately describe the data, as shown in Fig. S4a. The uncertainties reported in the main manuscript correspond to the standard deviations obtained from the covariance matrix of the fit, using absolute-σ weighting to properly account for the experimental error bars.

To avoid any bias from the choice of initial parameters, we employed a multistart fitting routine in which the bi-exponential model was fitted repeatedly to the same decay trace using 200 randomized initial parameter guesses spanning more than one order of magnitude in amplitudes, time constants, and offset. Among all fits, the parameter set with the lowest χ^2 value was selected. This procedure confirms that the extracted fast and slow spin relaxation times are robust and insensitive to the starting parameters, and the resulting best-fit values match those reported in our main text. The distribution of fitted time constants across all initial guesses, shown in Figs. S4b and S4c, further illustrates that the final results do not depend on the chosen starting parameters. Finally, we note that the measured spin lifetimes are highly sensitive to experimental conditions such as excitation power, local temperature, sample geometry, and sample amount, which explains the variation in reported values across different measurements on the same complex. However, the obtained spin lifetimes remain consistent within the same order of magnitude.

2 Theoretical considerations

2.1 Addressable ions in a spin measurement

In the following, we estimate the order of magnitude of ions that are resonantly driven by the RF field and optically readout in a single measurement, taking into account our experimental conditions such as sample geometry, pulse parameters, and measured signals.

We assume that the 200 µm core multimode fiber (NA = 0.5) is in contact with the flat surface of the macroscopic crystal, which is integrated into the Teflon ferrule. The propagation path is taken as the 500 µm thickness of the crystal, and the refractive index of the crystalline material is 1.5 [1]. The excitation volume through the crystal is approximated as a truncated cone with an initial diameter of 200 µm and an exit exit diameter of ∼400 µm, leading to an illuminated volume of $V \sim 10 \times 10^{-4}\,\mathrm{cm}^3$. Given the concentration of $C_{\mathrm{Eu}} = 9.6 \times 10^{20}\,\mathrm{ions/cm}^3$, the number of ions spatially located within the light cone is $\sim 1 \times 10^{17}$ ions. We account for the fraction of ions optically probed within the inhomogeneous line by $\eta_{\mathrm{h}} = \Gamma_{\mathrm{h}}/\Gamma_{\mathrm{inh}} \approx 1/23000$, the fraction of the $^{151}\mathrm{Eu}^{3+}$ isotope $\eta_{151} = 0.5$, and the fraction of ions within the spin inhomogeneous line driven by the RF field including the overall spin contrast $\eta_{\mathrm{s}} = 0.2 \times \Gamma_{\mathrm{s,h}}/\Gamma_{\mathrm{s,inh}}$, and obtain a probed ion number

$$N_p = C_{\mathrm{Eu}} V \eta_{\mathrm{h}} \eta_{151} \eta_{\mathrm{s}} \approx 10^{10}. \tag{S1}$$

With a collection efficiency of $\lesssim 1\,\%$, we thus detect signals corresponding to $\sim 10^8$ ions. Using a single photon counting module instead of an APD, about $10^5\times$ smaller signals can be recorded with similar signal to noise ratio. With improved photonic structures for higher collection efficiency, this will enable ODNMR on nanoscopic ensembles.

2.2 Optical dephasing

At a temperature of 4.2 K, the remaining homogeneous linewidth is dominated by phonon-induced dephasing arising from quasi-localized low-frequency vibrational modes, which follows an exponential temperature dependence. Below about 3.5 K, dephasing becomes governed by coupling to two-level systems, leading to an approximately linear temperature dependence of the linewidth of the linewidth. An extrapolation to zero temperature yields a finite residual dephasing rate, which can partly be attributed to ion-ion interactions causing instantaneous spectral diffusion. Additional, but comparatively minor, contributions may stem from spectral diffusion due to local magnetic and electric field fluctuations of the nuclear spin environment or paramagnetic impurities, and trapped charges, respectively. Studying these effects at millikelvin temperatures and diluted ion concentration will be an important next step towards maximizing the optical coherence.

2.3 Nuclear spin lifetime

The nuclear spin decay arises from multiple mechanisms, including phonon-mediated processes such as direct phonon, Raman, and Orbach processes, coupling to paramagnetic impurities, and energy exchange with two-level systems. The appearance of two time constants has been observed previously in many Eu^{3+}-based molecular complexes studied so far [2–4], as well as in solid-state crystals such Eu^{3+}:Y_2SiO_5 crystals [5]. First, the three hyperfine levels of both $^{151}Eu^{3+}$ and $^{153}Eu^{3+}$ isotopes exhibit different spin-lattice relaxation rates, so that SHB measurements addressing all hyperfine states potentially yield a multi-exponential decay. Another contribution may arise from a sub-ensemble of ions located near defects or paramagnetic impurities leading to a larger decay rate. Finally, the excitation of non-equilibrium vibrational modes within the molecular crystal induced during the SHB measurement may lead to an increased spin-lattice relaxation rate. Determining the dominant mechanism will require systematic temperature-, excitation power-, and Eu^{3+} doping concentration-dependent studies, which go beyond the scope of the present work.

2.4 Nuclear spin dephasing sources

One dephasing source for the europium nuclear spin is expected to be surrounding nuclear spins at the ligands. As discussed in the main manuscript, the stretching factors observed in the spin echo and CPMG decays already indicate a non-trivial bath. In order to extract a bath correlation time and a bath coupling strength, we initially assumed an Ornstein-Uhlenbeck model for the bath and fitted the decay of the visibility in the CPMG measurements according to the following relation [6]:

$$\overline{\rho}(N,\tau) = a \cdot \exp\left(-(\sigma\tau_c)^2 \left\{ \left[\frac{1}{\tau_c} - \frac{2}{\tau} \tanh\left(\frac{\tau}{2\tau_c}\right) \right] t - \left[1 + (-1)^{N+1} e^{-t/\tau_c} \right] \left[1 - \operatorname{sech}\left(\frac{\tau}{2\tau_c}\right) \right]^2 \right\} \right), \tag{S2}$$

where $t = N \cdot \tau$ represents the total evolution time, N denotes the number of applied refocusing pulses during the CPMG measurements, σ is the bath coupling strength and τ_c the bath correlation time. While the Ornstein-Uhlenbeck model provides a useful mathematical description for a single spin coupled to a spin bath of Gaussian fluctuations with a Lorentzian noise spectrum [7], recent theoretical work reported in [7–9] has shown that the decay of an ensemble of spins embedded in a dipolar-coupled environment is modified by averaging over the positional randomness of the probed spin ensemble. This averaging leads to a characteristic stretching factor of 1.5 instead of the value of 3 expected for a single spin coupled to a Lorentzian bath. In our case, the ensemble-averaged model is therefore more consistent with the measured decay behavior, and we include both perspectives below.

Using the ensemble relation reported in [8]:

$$T_2^{\text{ens}} = 2^{-1/3} \cdot T_{2,\text{s(Echo)}}^{\text{single}} \quad , \tag{S3}$$

where $T_{2,\mathrm{s(Echo)}}^{\mathrm{single}}$ is the decay time for a single spin of the probed ensemble, and our measured decay time $T_2^{\mathrm{ens}} = 0.61\,\mathrm{ms}$, together with average coupling strength $b = 12\,\mathrm{kHz}$ extracted from spin hole burning, the ensemble model yields a refined estimate of the bath correlation time of $\tau_B \approx 5.5\,\mathrm{ms}$. For comparison, fitting the same data within the single spin Ornstein-Uhlenbeck model results in a larger correlation time of about $\tau_B \approx 13\,\mathrm{ms}$. In our case, the distribution of bath spins in the molecular complex is not purely random, as the proton spins at the ligand fields occupy well-defined positions, while the ^{13}C spins remain randomly distributed. Additional contributions to the dephasing arise from paramagnetic impurities and phonons, which are expected to exhibit short or vanishing correlation times, can further modify the stretching factor. A more complete picture of the decoherence mechanism could be obtained from microscopic simulations that integrate over the spatial distribution of all bath spins in the molecule, following the approaches introduced in [7, 9]. Such modeling, which would capture the full ensemble-averaging effects, can be complemented with measurements of the temperature dependence of the spin coherence.

To assess whether the value of the bath coupling strength obtained from spin hole burning is consistent with a bath of nuclear spins located on the ligands, we calculate the interaction strength between a single europium nuclear spin and a hydrogen nuclear spin. The interaction strength is given by:

$$E = \frac{\mu_0}{4\pi} \cdot \frac{\mu_{\mathrm{Eu}}\mu_{\mathrm{H}}}{r^3}. \tag{S4}$$

The molecular complex features 48 hydrogen atoms and a nearby nitrogen atom ($\sim$4 Å), which is located in the counter ion. The distance for the hydrogen atoms ranges between 4 Å to 8 Å. The gyromagnetic ratio of europium is $6.65 \times 10^7\,\mathrm{rad\,s^{-1}\,T^{-1}}$ (I = 5/2) and for a proton $2.68 \times 10^8\,\mathrm{rad\,s^{-1}\,T^{-1}}$ (I = 1/2), leading to an interaction strength of $\approx$72 Hz at 8 Å and $\sim$583 Hz at 4 Å. Both calculated coupling values are small compared to the extracted 12 kHz bath coupling strength, and also the total field of a randomly oriented nuclear spin ensemble is expected to lead to a smaller coupling.

As an additional noise source, other REI species originating from impurities in the precursor materials can exhibit an electron spin magnetic moment that would couple stronger to the europium nuclear spin. For an electron spin contribution, we assume the magnetic moment of μ_{B} (g-factor of 2). The formula S4 can be rearranged to solve for the distance r. By inserting the coupling strength of $\approx$12 kHz into the equation, we obtain a distance of $\approx$13 Å. This resulting distance is comparable to the separation of neighboring Eu centers within the unit cell, which range from 9.35 Å to 10.40 Å. However, the impurity concentration of the europium chloride used for synthesizing the molecular complex was about 99.99 %, and average impurity distances are expected to be $\sim$20 nm. Impurities are thus not expected to dominate the dephasing.

Finally, quasi-local low-frequency vibrational modes [10] have been observed to affect the optical homogeneous linewidth in the studied complex above 3.5 K [1]. Such vibrations can modulate the local electric field gradient at the Eu nucleus, which affects the ligand field contribution to the quadrupole splitting. This is expected from the observed correlation of the spin and optical transition frequency. Already a moderate reduction in temperature may significantly reduce this contribution.

2.5 Correlation between optical and spin inhomogeneous lines

Quadrupolar Hamiltonian and expected optical-RF correlation

As explained in the main text, a linear approximation is not precise for our case, and a quadratic dependence is expected [11]. This follows from the quadrupolar nuclear spin Hamiltonian:

$$H = P\left(I_z^2 - \frac{1}{3}I(I+1) + \frac{\eta}{3}(I_x^2 - I_y^2)\right), \tag{S5}$$

where $P = const. \cdot V_{zz}/(I(I-1))$ is the effective nuclear electric quadrupole interaction constant, $\eta = (V_{xx} - V_{yy})/V_{zz}$ is the electric field asymmetry parameter, and V_{ii} are the components of the tensor of the electric field gradient at the nucleus. Due to J-mixing induced by the ligand field, the optical transition frequency acquires contributions from higher J levels. Since the same ligand field also determines the hyperfine structure, perturbation theory provides a direct link between the optical and RF transition energies.

Comparison with solid-state materials

Yamaguchi et al. studied REI-doped solid-state materials, in particular Eu^{3+}:$YAlO_3$ [11], where the following assumptions were made:

- Hyperfine splittings differ by a factor of two in case of the $^{151}Eu^{3+}$ isotope,
- Electric-field gradient asymmetry parameter $\eta \approx 0$,
- Simplified Hamiltonian yields a direct dependence on the spin transition frequency δ_{RF} to P: $\delta_{\mathrm{RF}} = 2P$.

These assumptions lead to a quadratic dependence:

$$E_{\mathrm{opt}} = E_0 + c \cdot \delta_{\mathrm{RF}}^2 \quad , \tag{S6}$$

which is further approximated to a linear and positive dependence for small variations of δ_{RF}.

In contrast, our examined Eu^{3+}-based molecular complex shows ground state hyperfine splittings of 21.5 MHz and 34 MHz for the $^{151}Eu^{3+}$ isotope, in agreement with our earlier measurements in [1]. This results in a ratio of 1.58, and the asymmetry parameter is $\eta = 0.47$. Furthermore, the point group symmetry around the Eu^{3+} center is C_{2v} compared to C_s in [11], which implies different crystal-field parameter constraints. While $|B_{21}|$ is zero in both cases, the $|B_{22}|$ parameter also vanishes for C_s symmetry in [11] but is non-zero for C_{2v} symmetry [12], which applies to our molecule.

Together with the non-zero asymmetry parameter η, these differences in the crystal-field contributions affect the optical and spin transitions in our case and can lead to different magnitudes and opposite signs in their correlation gradient, explaining the reversed slope observed in our measurements.

Role of strain and inhomogeneous broadening

The main optical inhomogeneous linewidth reported in [11] is below 2 GHz, similar to the linewidth we observe in our slowly grown crystal, which exhibits $\Gamma_{\mathrm{inh}} = 1.94$ GHz.

However, the crystal used for spin characterization features a significantly larger linewidth of $\Gamma_{\mathrm{inh}} = 23\,\mathrm{GHz}$, likely caused by increased strain arising from the faster growth process or from mechanical forces during device integration. This larger inhomogeneous broadening corresponds to a wider variation range of the parameters P and η, which reduces the validity of a linear approximation and leads to deviations from the behavior described in [11].

3 Figures

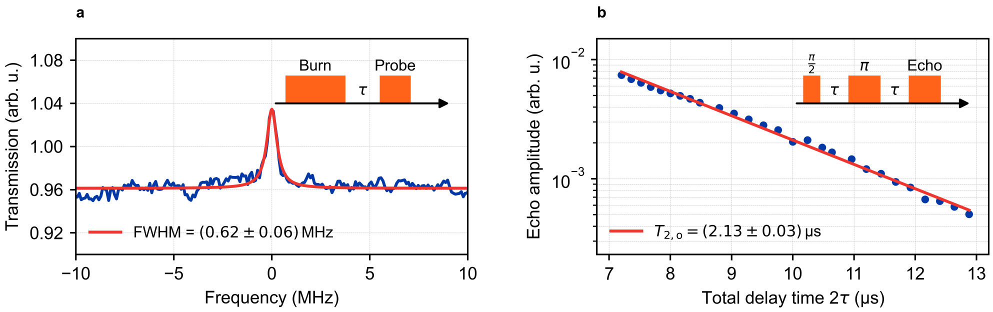

Fig. S1 Characterization of optical properties. (a) A spectral hole burnt into the inhomogeneous profile of crystal 2 yields a hole linewidth of 620(60) kHz. **(b)** Photon echo amplitude decay as a function of the total delay time 2τ, resulting in an optical coherence time $T_{2,\mathrm{o}}$ of 2.13(3) µs. The echo signal was measured using heterodyne detection. Both measurements were performed at a temperature of 4.2 K.

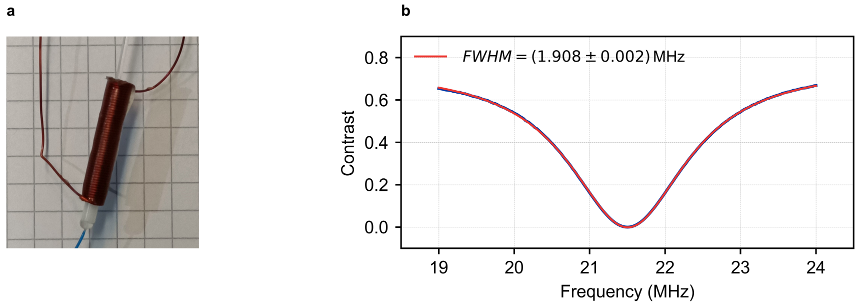

Fig. S2 Resonance characterization of the RF coil. (a) A photograph of the experimental setup featuring both excitation (blue jacket) and collection fiber (transparent) covered by the RF coil with compact winding geometry. The coil is encapsulated by an epoxy adhesive to ensure mechanical robustness. **(b)** Reflection spectrum of the same coil, tuned to 21.5 MHz. A Fano resonance fit is applied to extract the FWHM of ∼1.9 MHz, yielding a quality factor Q∼11.

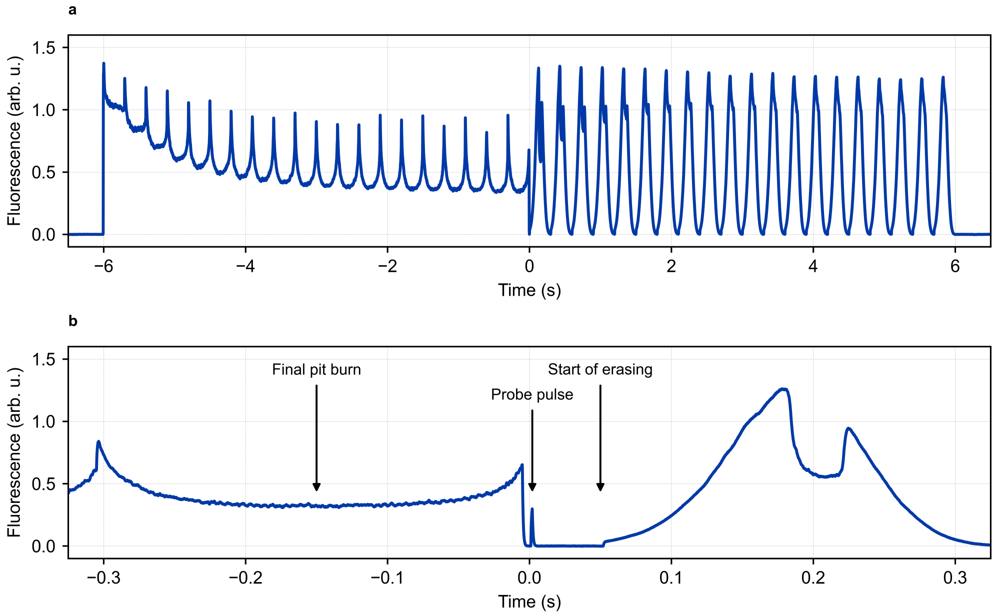

Fig. S3 Spectral pit preparation. (a) Full time trace of one exemplary spectral pit preparation sequence. **(b)** Zoom into the spectral pit preparation sequence showing the final pit burn, the short probe pulse and the start of the erasing sequence.

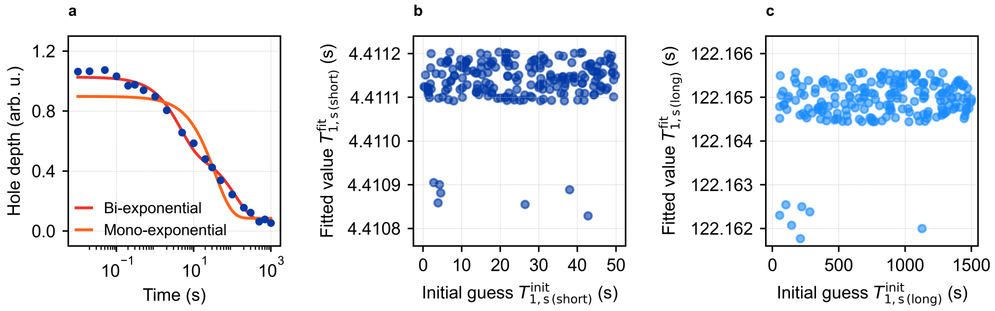

Fig. S4 Mono- vs. bi-exponential fit and robustness analysis. (a) Comparison of the mono-exponential and bi-exponential fits to the spin lifetime decay shown in Fig. 2b. The mono-exponential model does not adequately represent the data. **(b,c)** Distribution of the fitted fast and slow time constants obtained from 200 randomized initial parameter guesses. The narrow clustering around the best-fit values demonstrates that the extracted time constants are robust and insensitive to the choice of initial parameters.

4 Tables

Table S1 Coil geometry.

Parameter	Used coil
Number of turns	62
Inner diameter (mm)	2.55
Outer diameter (mm)	3.45
Length (cm)	2

References

[1] Serrano, D., Kuppusamy, S.K., Heinrich, B., Fuhr, O., Hunger, D., Ruben, M., Goldner, P.: Ultra-narrow optical linewidths in rare-earth molecular crystals. Nature **603**, 241–246 (2022) https://doi.org/10.1038/s41586-021-04316-2

[2] Kuppusamy, S.K., Serrano, D., Nonat, A.M., Heinrich, B., Karmazin, L., Charbonnière, L.J., Goldner, P., Ruben, M.: Optical spin-state polarization in a binuclear europium complex towards molecule-based coherent light-spin interfaces. Nature Communications **12**, 2152 (2021) https://doi.org/10.1038/s41467-021-22383-x

[3] Kuppusamy, S.K., Vasilenko, E., Li, W., Hessenauer, J., Ioannou, C., Fuhr, O., Hunger, D., Ruben, M.: Observation of narrow optical homogeneous linewidth and long nuclear spin lifetimes in a prototypical [eu(trensal)] complex. The Journal of Physical Chemistry C **127**(22), 10670–10679 (2023) https://doi.org/10.1021/acs.jpcc.3c02903 . Publisher: American Chemical Society. Accessed 2023-06-16

[4] Kuppusamy, S.K., Hunger, D., Ruben, M., Goldner, P., Serrano, D.: Spin-bearing molecules as optically addressable platforms for quantum technologies. Nanophotonics **13**(24), 4357–4379 (2024) https://doi.org/10.1515/nanoph-2024-0420 . Publisher: De Gruyter. Accessed 2025-07-24

[5] Könz, F., Sun, Y., Thiel, C.W., Cone, R.L., Equall, R.W., Hutcheson, R.L., Macfarlane, R.M.: Temperature and concentration dependence of optical dephasing, spectral-hole lifetime, and anisotropic absorption in eu^{3+}:y_2sio_5. Physical Review B **68**, 085109 (2003) https://doi.org/10.1103/PhysRevB.68.085109

[6] Pascual-Winter, M.F., Tongning, R.-C., Chanelière, T., Le Gouët, J.-L.: Spin coherence lifetime extension in Tm^{3+}:YAG through dynamical decoupling. Physical Review B **86**, 184301 (2012) https://doi.org/10.1103/PhysRevB.86.184301 . (Received 8 August 2012; published 6 November 2012)

[7] Bauch, E., Singh, S., Lee, J., Hart, C.A., Schloss, J.M., Turner, M.J., Barry, J.F., Pham, L.M., Bar-Gill, N., Yelin, S.F., Walsworth, R.L.: Decoherence of ensembles of nitrogen-vacancy centers in diamond. Physical Review B **102**, 134210 (2020) https://doi.org/10.1103/PhysRevB.102.134210

[8] Davis, E.J., Ye, B., Machado, F., Meynell, S.A., Wu, W., Mittiga, T., Schenken, W., Joos, M., Kobrin, B., Lyu, Y., Wang, Z., Bluvstein, D., Choi, S., Zu, C., Bleszynski Jayich, A.C., Yao, N.Y.: Probing many-body dynamics in a two-dimensional dipolar spin ensemble. Nature Physics **19**, 836–844 (2023) https://doi.org/10.1038/s41567-023-01944-5

[9] Marcks, J.C., Onizhuk, M., Delegan, N., Wang, Y.-X., Fukami, W., Watts, M., Clerk, A.A., Heremans, F.J., Galli, G., Awschalom, D.D.: Guiding diamond spin qubit growth with computational methods. Physical Review Materials **8**, 026204

(2024) https://doi.org/10.1103/PhysRevMaterials.8.026204

[10] Kozankiewicz, B., Orrit, M.: Single-molecule photophysics, from cryogenic to ambient conditions. Chemical Society Reviews **43**(4), 1029–1043 (2014) https://doi.org/10.1039/C3CS60165J . Publisher: The Royal Society of Chemistry. Accessed 2025-08-12

[11] Yamaguchi, M., Koyama, K., Suemoto, T., Mitsunaga, M.: Perturbed ion sites in Eu^{3+} : $YAlO_3$ studied by optical-rf double-resonance spectroscopy. Physical Review B **59**(14), 9126–9131 (1999) https://doi.org/10.1103/PhysRevB.59.9126 . Publisher: American Physical Society. Accessed 2025-08-01

[12] Tanaka, M., Nishimura, G., Kushida, T.: Contribution of j mixing to the $^5d_0{-}^7f_0$ transition of eu^{3+} ions in several host matrices. Phys. Rev. B **49**, 16917–16925 (1994) https://doi.org/10.1103/PhysRevB.49.16917
